# The Influence of Coordinative Skills on the Oral Health of Children and Adolescents in Permanent Dentition

**DOI:** 10.3390/jcm11216472

**Published:** 2022-10-31

**Authors:** Henrike Kolbow, Wieland Kiess, Christian Hirsch, Mandy Vogel, Annett Schrock, Wieland Elger

**Affiliations:** 1 Department of Pediatric Dentistry, University of Leipzig, 04103 Leipzig, Germany; 2 LIFE Leipzig Research Center for Civilization Diseases, University of Leipzig, 04103 Leipzig, Germany; 3 Department of Women and Child Health, Hospital for Children and Adolescents and Center for Pediatric Research, University of Leipzig, 04103 Leipzig, Germany

**Keywords:** oral prophylaxis, oral health, children, coordinative skills, dental plaque, toothbrushing

## Abstract

Removing dental plaque by using a toothbrush is the most important measure for oral hygiene. The aim of the present study was to estimate the impact of the coordination skills of children and adolescents on their oral health (plaque level, DMF/T: decayed, missing, filled teeth). Within a prospective cohort study, 996 children (10 to 18 years) were examined. The results of three coordination tests from the Motorik Modul (MoMo) were included to evaluate the coordination skills. Other parameters taken into account were age, sex, orthodontic treatment and socioeconomic status (SES). Univariate and various multivariate analyses were performed to evaluate relationships. Better results in precision coordination tests were significantly related to a better oral hygiene (backward balancing: logistic regression OR 0.86, 95%CI: 0.73–0.99, *p* = 0.051, proportional odds model OR 0.86, 95%CI: 0.75–0.99, *p* = 0.037; one-leg-stand: logistic regression OR 0.78, 95%CI: 0.63–0.96, *p* = 0.018, proportional odds model OR 0.77, 95%CI: 0.64–0.92, *p* = 0.003). Higher scores on one-leg-stand were significantly related to a lower caries prevalence (logistic regression OR 0.81, 95%CI: 0.66–0.99, *p* = 0.037; Poisson regression exp(ß) 0.82, 95%CI: 0.74–0.91, *p* < 0.001). Coordination test under a time constraint (jumping side-to-side) showed no significant relation. Oral hygiene was poorer in younger children, boys and low SES. Caries prevalence increased with low SES and increasing age. The present results suggest that oral health is influenced by coordinative skills.

## 1. Introduction

Poor oral hygiene can result in tooth decay and loss, gingivitis, periodontitis, halitosis and gum diseases [1]. This can negatively affect one’s quality of life, social functioning, school performance, physical and psychological health and one’s economic opportunities [2,3]. One of the most prevalent chronic diseases in human populations is dental caries. It is the most common chronic disease in children [4,5]. In 2016, children in Germany at the age of 12 had a caries prevalence of 28.3% [6]. Understanding the determinants of oral health is important to enhance and develop interventions to prevent oral diseases. Early oral prophylaxis is important as adult oral health is predicted not only by childhood socioeconomic advantage or disadvantage, but also by oral health in childhood [7,8].

To reduce the accumulation of plaque, mechanical tooth cleaning is the mainstay [9,10,11]. Proper toothbrushing is a simple motor activity that can promote oral health by removing dental plaque [2]. During toothbrushing, the hand motion is controlled by the shoulder and wrist, while the elbow generates the cyclic rhythm [12]. The effect of brushing instructions could be evaluated not only by the efficiency of plaque removal but also by muscle activity [13]. Guidelines recommend supporting and supervising toothbrushing until children are 8 to 10 years old [14].

According to scientific research, adequate toothbrushing depends on the development of psychomotor skills, the child’s hand function and coordination [15,16]. Furthermore, the quality of toothbrushing depends on brushing methods including manual or powered toothbrushing and on the motor performance of the toothbrushing motion [17,18,19]. However, no technique of toothbrushing has been shown to be unequivocally more efficient than others [11]. We also know that oral health education alone does not improve toothbrushing skills or behaviours significantly [10,20].

Good oral hygiene presents a challenge for children because of their restricted motor skills as the use of a toothbrush is the most important measure for good oral hygiene [21]. It has been shown that children with motor coordination disorders and level of motor ability (developmental coordination disorder) have poorer performance regarding daily oral hygiene [22]. We also know that motor development deficits observed in early childhood are still apparent in adolescence [23]. This and the persistent high prevalence of caries indicates that toothbrushing performances still need further intervention such as focusing on coordinative skills.

The present study fills the research gap on how coordinative skills influence children’s ability of toothbrushing by assessing the children’s coordinative skills as well as their oral health. We hypothesized that children with better coordination skills also have better oral health.

## 2. Materials and Methods

### 2.1. Participants and Data Collection

For the present analysis, data from the LIFE Child study (clinical trial no. NCT02550236) were analyzed. The LIFE Child study is a large population-based cohort study, taking place in Leipzig, Germany [24]. The study aims to investigate how genetic, metabolic and environmental factors can influence the health and development of children and adolescents. The study was designed in accordance with the declaration of Helsinki and under the supervision of the Ethics Committee of the Medical Faculty of the University of Leipzig (Reg. No. 264/10-ek). The study participants were recruited through a network of hospitals, health centers, schools and kindergartens. Apart from children with chronic, chromosomal and syndromic diseases, all children were able to participate in the LIFE Child study. Before the start of the study, children and parents were informed about the aim and procedure of the study and asked for written consent [25,26].

We decided to only include children older than 9 years because we wanted participants to brush their teeth independently. In the current research, participants aged 10 to 18 years were studied, considering only one visit of each child from 2011 to 2015. During this time, participants underwent various medical, dental and motor examinations. Additional information was obtained through questionnaires [27]. Privacy protection was ensured by anonymizing the data, as described elsewhere [24,25].

### 2.2. Coordination Tests

To assess the children’s motor performance, a test profile based on the Motorik-Modul (MoMo) Longitudinal Study was used. MoMo is part of the German Health Survey for Children and Adolescents (KiGGS) of the Robert Koch Institute, further described elsewhere [28]. By measuring cardiorespiratory fitness, strength, coordination, speed and mobility motor abilities were systemized [29]. The test profile consisted of 11 items [30]. The modular character of the MoMo allows researchers to use either the whole test module, single tests or a sample of numerous test items.

In the LIFE Child study, coordinative abilities were assessed by 3 different tests. Backward balancing and one-leg-stand estimated motor coordination. Focusing on dynamic precision, the task jumping side-to-side evaluated motor coordination under time constraint. All tests were accomplished as described by Woll et al. by trained and certified study examiners in a sports room at the Leipzig Research Center for Civilization Diseases [31].

The one-leg-stand was used to check large motor coordination in static precision tasks and was adopted from the screening test for the school enrollment of Schilling and Baedtke [32]. The participants were instructed to stand on their dominant leg for 1 min with their eyes open and the number of floor contacts with the other leg was counted. The test balancing backwards is based on the Hamm–Marburg Körperkoordinationstest (KTK) for children and is used to check the dynamic balance of the whole body [33]. For testing the backwards balancing skills, participants were required to balance backwards on three beams of different latitudes, having two attempts per beam. The numbers of steps were counted (up to eight steps per attempt) and added up to a final score. If a participant’s foot or hand touched the ground or wall, the attempt was finished [34]. The jumping side-to-side test, also based on KTK, is used to record the speed of action and endurance of the lower extremities and examinates large motor coordination under time pressure. In the side-to-side jumping test, attendees were asked to jump side-to-side from one mark painted on the ground to an adjacent mark for 15 s. The final score was calculated from the average of two trials.

### 2.3. Sociodemographic Predictors

Socioeconomic status (SES), sex and age were included as determinants in the analysis. SES was categorized into three categories: low, middle and high, and assessed by a parent questionnaire including the parent’s education and occupational qualification, occupational status and total net income of the family household [35]. The so-called Winkler Index calculated from this information ranges from 3 to 21, with higher values indicating a higher SES.

### 2.4. Clinical Examinations

Based on the method of Greene and Vermillion, oral hygiene was measured by the level of plaque [36]. Three categories were applied: 1 = good, no plaque or calculus; 2 = fair, localized plaque and/or calculus; 3 = poor, generalized presence of plaque and/or calculus. To assess the children’s caries experience in permanent dentition, the DMF/T-Index was measured at up to 28 teeth without consideration for third molars. A tooth was classified as decayed (D) in case of the presence of an open cavitation. Initial caries lesions were not considered. In case of tooth decay, teeth were registered as missing (M) and filled (F) teeth. The examination of the DMF/T-Index was carried out according to WHO criteria from the year 1997 [24].

Trained professionals performed dental examinations at the study center under standardized conditions. Each examiner was briefed prior to the investigation and was trained at least twice. To assess the reproducibility of the results, the intraclass correlation was calculated and reached an average value of >0.7, which corresponds to very high reproducibility. This quality check was repeated every 6 months or when a new examiner was introduced. The clinical examinations were performed at the study center using a standard examination chair. The data were obtained with the help of electronic case report forms [27].

### 2.5. Statistical Analysis

Statistical analyses were conducted using R (version 4.2.1, Vienna, Austria) and IBM SPSS Statistics (Version 27.0.1.0, IBM Corp., Armonk, NY, USA) [37]. The significance level was set to α = 0.05. Descriptive statistics are presented as means and standard deviation for continuous and counts and percentages for categorical variables. For the comparison between two groups, we used *t*-tests for continuous and χ2 tests for categorical variables. The three coordinate skills were transformed into age- and sex-adjusted standard deviation scores using German references [28]. DMF/T and OH were dichotomized into DMF/T = 0 (no caries experience) and DMF/T > 0 resp. into OH = 1 (no or little plaque) and OH > 1. Subsequently, the associations between the resulting binary variables as outcomes and the three coordinative skills as predictors were assessed using univariate and multivariate logistic regression analyses. Furthermore, Poisson regression was applied to assess the association between the degree of DMF/T as an outcome and each coordinative skill as predictor. Proportional odds regression models were used to assess the association between the three-level variable OH and each coordinative skill.

All multivariate models were adjusted for age, gender, SES and orthodontic treatment. The results were presented as odds ratios (OR) for logistic and proportional odds models and ratios (R = exp(β)) for Poisson regression models, including the 95% confidence interval. Finally, we estimated how much of the effect of SES was mediated by coordinative skills using a mediation analysis, implemented as structural equation models.

## 3. Results

### 3.1. Study Population

The study population included 996 children and adolescents, aged 10–18 years, and considered only one visit of each participant (Table 1 and Table 2). The mean age was 12.7 years, and with 50.8% girls both genders were represented almost equally. The population was characterized by a rather high SES as only 13.1% of the subjects were assigned to a low SES. In the past or at the time of examination, 23.1% of the participants underwent orthodontic treatment. As can be seen, 31.1% of the population had caries experience, meaning permanent dentition. Plaque was detected in 75.5% of the subjects.

### 3.2. Multivariate Analyses

**Figure 1:**
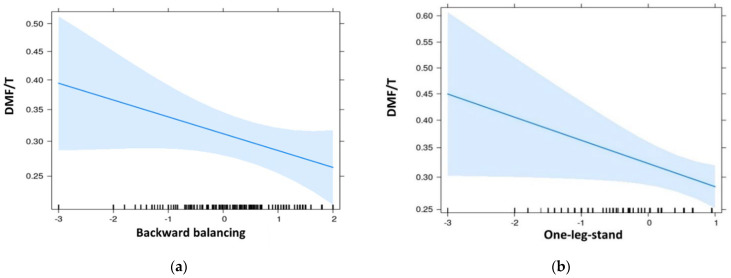
Effect plots of logistic regression analysis of DMF/T and coordination skills in children and adolescents of 10 to 18 years (Leipzig, Germany): (**a**) of the backward balancing test; (**b**) of the one-leg-stand test.

The results of the logistic regression analysis (Table 3) showed a significant relation between the children’s level of plaque (OR = 0.78, *p* = 0.018) and DMF/T (OR = 0.81, *p* = 0.037) and the results of the precisions coordination test, one-leg-stand (Figure 1). Good results in backward balancing were associated with better oral hygiene (OR 0.86) and almost reached the level of significance (*p* = 0.051).

The test of jumping side-to-side was the only test for which no significant association with either the level of plaque or DMF/T was found. A significant gender difference was observed for oral plaque accumulation (*p* = 0.001–0.008), with girls having a lower risk for having dental plaque (OR = 0.57) than boys. A lower SES was significantly related to higher plaque levels (OR 2.18–2.58, *p* = 0.001–0.008) and, in part significantly, to higher caries risk (OR = 1.65–1.86, *p* = 0.032–0.082). With increasing age, the risk of caries increased by approximately 25% at each year (*p* < 0.001), while oral hygiene improved (OR ≈ 0.9, *p* = 0.004–0.009). The potential influence of past orthodontic treatments on DMF/T was evaluated. Analysis also included whether wearing braces at the time of the study had an impact on oral hygiene. Logistic regression analysis revealed no significant interactions between orthodontic treatments and plaque accumulation or DMF/T.

**Figure 2:**
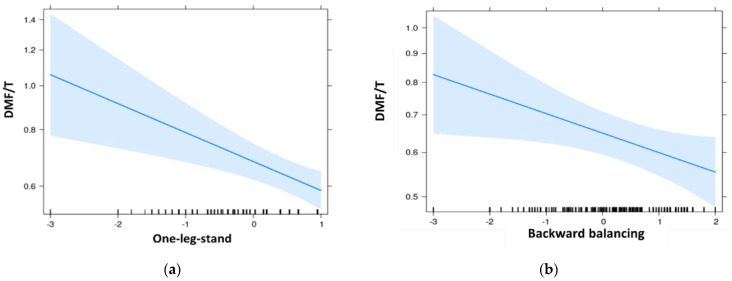
Effect plots of Poisson regression analysis of DMF/T and coordination skills in children and adolescents of 10 to 18 years (Leipzig, Germany): (**a**) of the backward balancing test; (**b**) of the one-leg-stand test.

The results of the Poisson regression analysis (Table 4, Figure 2) showed that the DMF/T decreased by a factor of 0.82 when test results were better in the one-leg-stand. (*p* < 0.001). Caries experience increased with age (exp (ß) ≈ 1.2, *p* < 0.001), low SES (exp (ß) = 2.31–2.50, *p* < 0.001), and increased plaque accumulation (exp (ß)_fair_ = 2.16–2.20, *p* < 0.001; exp (ß)_poor_ = 4.35–4.57, *p* < 0.001). No significant relation between the coordinative balancing backwards tests and jumping side-to-side and DMF/T could be found. In addition, we found no evidence of association between subjects’ sex and their caries experience. Adjusted for backward balancing, past orthodontic treatment had a significant effect on caries prevalence. For one-leg stand and side-to-side jumps, the significance level was also almost reached.

**Figure 3:**
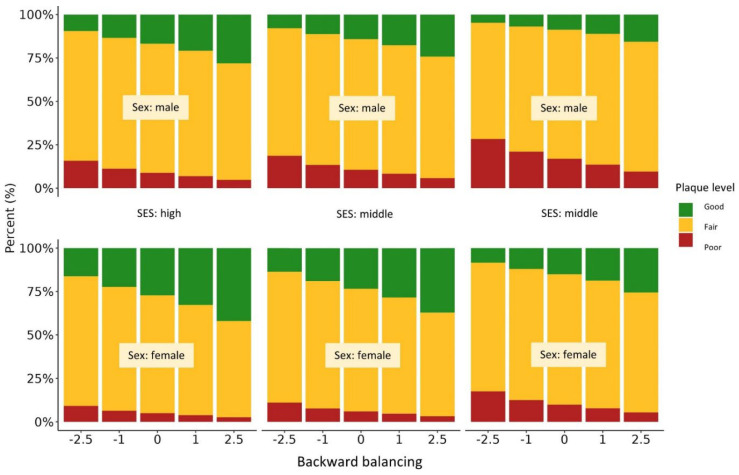
Proportional odds model of oral hygiene and the coordination backward balancing test.

**Figure 4:**
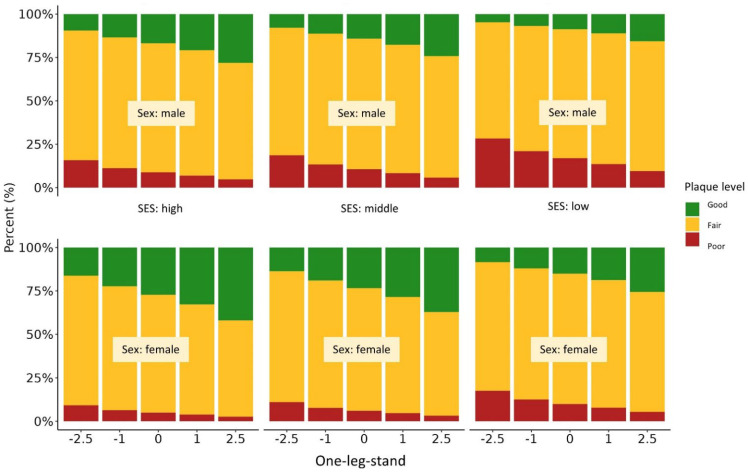
Proportional odds model of oral hygiene and the coordination one-leg-stand test.

**Figure 5:**
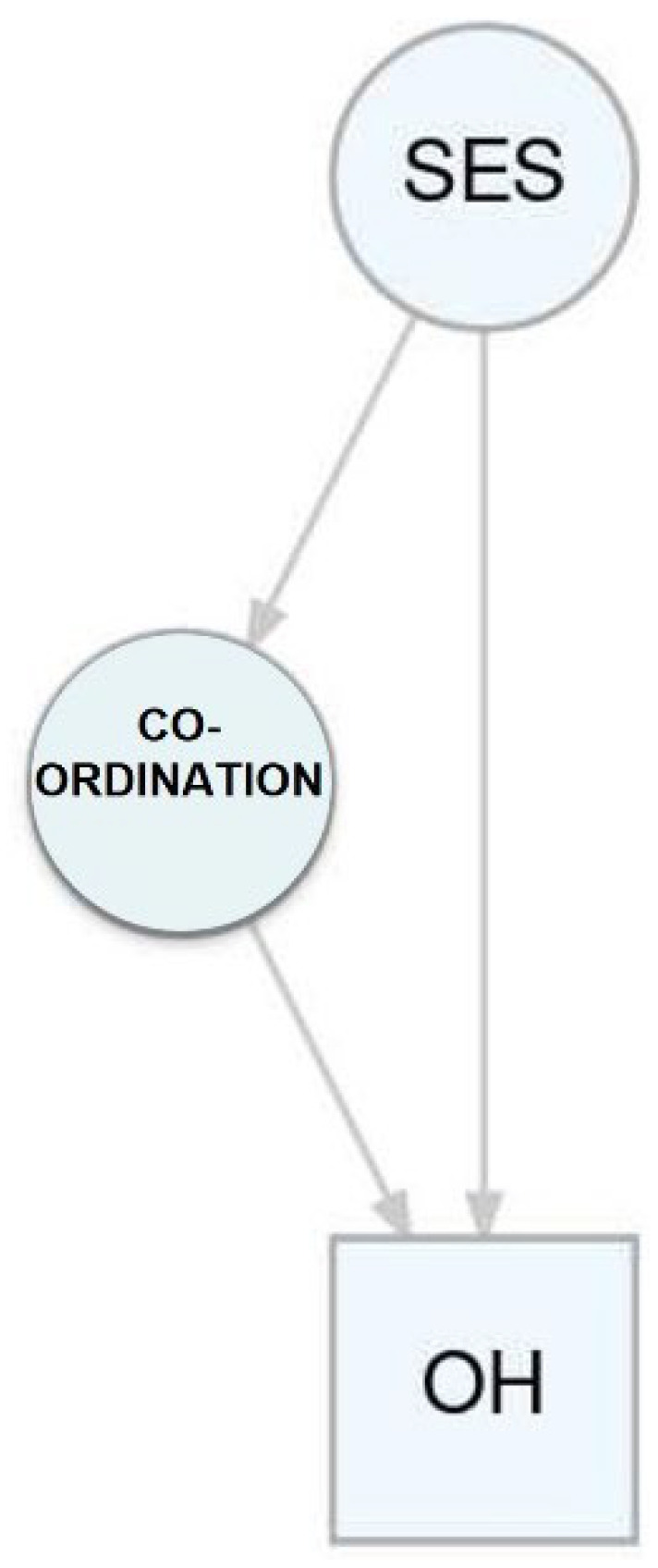
Mediation analysis of oral hygiene (OH), SES and coordinative abilities.

As revealed by the proportional odds model, better results in precision coordination tests (one-leg-stand: *p* = 0.003; backward balancing: *p* = 0.037) were significantly related to better oral hygiene (Table 5, Figure 3 and Figure 4). The odds for more plaque accumulation were reduced by about 23% when having better results in the one-leg-stand test. For better scores in the backwards balancing test, a risk reduction for dental plaque by about 14% was shown. The side-to-side jumping test showed no significant association with the level of plaque (Figure 5). A low SES doubled the odds for more dental plaque (OR = 2.1–2.4, *p* = < 0.001–0.002). With increasing age, the level of plaque accumulation decreased (OR ≈ 0.9, *p* = 0.014–0.023). In addition, the analysis revealed that girls in our cohort had odds ratios of approximately 0.5 for dental plaque compared with boys (*p* < 0.001). Again, we found no significant influence of orthodontic treatments on the participants’ oral health. The proportional odds model provided a useful extension of the binary logistic models as the response variable dental plaque is defined in ordered categories [38].

### 3.3. Mediation Analysis

Approximately 20% of the effect of SES was mediated by the coordinative one-leg stand test (*p* = 0.004); balancing backwards mediated 14% of the effect (*p* = 0.032) (Figure 5). No corresponding effect could be demonstrated for the jumping side-to-side test. There was no evidence that the coordinative ability mediates the effect of the SES on the DMF/T.

## 4. Discussion

The present study investigated whether better coordinative skills are associated with better oral health in children and adolescents and if this persists adjustment for SES. To the authors’ knowledge, this is the first study to focus on children’s and adolescents’ coordinative abilities in direct association with their oral health. In this context, we were able to show that oral health in terms of oral hygiene (plaque level) as well as caries prevalence (DMF/T) is very well associated with, among other parameters, coordinative abilities (backward balancing, one-leg-stand). This relationship was also indicated in other studies that revealed limited motor and fine motor skills as restrictive factors for oral health [21,22,39]. With rising age, oral hygiene improved, but the caries index increased. Girls had lower plaque levels both univariately and in multivariate analyses. However, there was no sex difference with respect to DMF/T. Subjects with low socioeconomic status were both more likely to have fair/poor oral hygiene and higher caries prevalence. Orthodontic therapy, whether in the past or at the time of the examination, showed no influence except for a significantly reduced caries risk for children with better results in the backward balancing test.

In the present study, about 75% of the subjects examined had fair or poor oral hygiene. This appears somewhat higher than in the study by Diamanti et al. who examined a comparable age cohort [40]. However, it is still in a similar range, with 21.5% of 12-year-olds and 37.2% of 15-year-olds having good oral hygiene in the above study. The phenomenon of better oral hygiene with increasing age is also evident in our analysis. A similar effect was also found by Kudirkaite et al., and it seems plausible that brushing skills and awareness of good oral hygiene are increasing in adolescents [41]. Approximately 70% of the study participants had no caries experience at the defect level. In a national study, 12-year-olds from the studied region had a slightly lower value of about 20% defect caries [6]. However, we also studied older adolescents, and it can be assumed that caries prevalence increases with age. The number of carious lesions is subject to a cumulative effect and must therefore statistically inevitably grow. In another German national study, only 2.5% of young adults had no caries at all [42]. That girls seem to brush their teeth better or/and more often was shown in each of our analyses and is also confirmed by other studies [41,43]. However, this does not result in a significant difference in the DMF/T index, which is in line with the results of the German Oral Health Study V [42]. One explanation for this could be the multifactorial nature of caries development. Various parameters must be taken into consideration and not only brushing is of importance. Nevertheless, our analyses confirmed that plaque accumulation was still positively associated with DMF/T.

It is scientific consensus that oral health is strongly determined by social factors, and this is also applicable in Germany [7,8,40,41,42]. Our findings are in line with this, as we were able to confirm this in each of our analyses. SES was a distinct factor, and subjects from a lower socioeconomic household had poorer scores in both oral health parameters compared with the middle or high class. We also know that coordinative abilities are dependent on SES [28]. As revealed by mediation analysis, the effect of SES on oral hygiene was mediated by coordinative skills of the subjects. From this, we can conclude that promoting coordination in vulnerable groups (e.g., low SES) might improve their oral health. These motor skills thus also appear to be of paramount importance. Even after adjusting for all possible confounders mentioned so far, significantly less plaque or calculus was found and lower DMF/T values were determined when coordination skills were better.

Our study results were consistent with our hypothesis. Children with better precisions skills according to the one-leg-stand and side-to-side jumping tests had less plaque. In our opinion, these two tests can draw conclusions about individuals’ ability to brush their teeth. On the one hand, the structure of the “Motorik-Modul” allows a selection of the tests without losing their meaningfulness [28]. On the other hand, the process of brushing teeth is coordinative [15,44]. The side-to-side jumping test showed in none of the models to have a positive association with the plaque level or DMF/T. A possible reason for this finding is that the test measures the speed of action and the local strength endurance of the lower extremities more than the coordinative component [33]. The DMF/T correlated only with the one-leg-stand; no association with the backward balancing or jumping side-to-side test was found. We expected less correlation between the coordinative test and DMF/T because of the limitations of the DMF/T. The discrepancy between the incidence of plaque accumulation and DMF/T is caused by the pathogenesis of caries. Cariogenic bacteria of dental plaque need time and substrate to cause irreversible demineralisation [45]. Because of that, we evaluated the level of plaque as a more valid variable to assess oral health within our study. In addition to that, the study cohort was characterized by a rather high SES, which must be considered for the interpretation of all results. In addition, we assumed, because of the stable level of oral health as well as motor abilities during growing up, that our results are transferable to children of younger age [7,23].

Based on the knowledge of the pathogenesis of dental caries, future studies should include the children’s diet as well as the frequency of dental check-ups as these predictors influence oral hygiene [46,47]. In addition, no questions were asked about the participant’s fluoride intake. The daily use of fluoride is the most evidence-based approach to prevent dental caries and, therefore, an important determinant for caries prevalence [48]. Bös et al. evaluated the coordinative skills with a further test for fine motor skills, which might be a good extension for future investigations [28]. Still, the large sample size of the cohort and the broad age range is a large strength of the study.

Orthodontic treatment of the subjects at the time of the investigation had no effect on the plaque level. According to Kudirkaite et al., orthodontic appliances increase plaque accumulation [41]. We did not observe this in our cohort. Regarding the relationship between previous orthodontic treatments and DMF/T, only Poisson regression, including the backward balancing test, showed a significant effect. A current review from Müller et al., a national survey by Choi et al. and a prospective cohort study by Cave et. al. showed no higher caries risk because of orthodontics [1,49,50]. Walsh et al. formulated only an increased caries risk due to orthodontic appliances [51]. A possible reason for our results differing from current study results may be the small number of children with orthodontic appliances. This is an unavoidable result of the population-based (and not orthodontic or dental) background of the LIFE Child study.

## 5. Conclusions

The present findings indicate that the children’s ability for oral care is influenced by their coordinative skills. The oral health of children and adolescents is influenced by multiple factors. Therefore, oral prophylaxis should be developed in an interdisciplinary manner and include coordinative support. Furthermore, research is necessary.

## Figures and Tables

**Table 1:** Association of oral hygiene and SES, sex, age, orthodontic treatment, DMF/T and coordination skills in children and adolescents of 10 to 18 years (Leipzig, Germany).

	Oral Hygiene		Total	*p*-Value
	Good	Fair/poor		
Total	24.5% (N = 244)	75.5% (N = 752)	100% (N = 996)	
Mean age (±SD) yrs. ^++^	13.05 (±2.16)	12.63 (±2.06)	12.73 (±2.09)	0.007
Sex % (N) *				
Male	38.5 (94)	52.7 (396)	49.2 (490)	
Female	61.5 (150)	47.3 (356)	50.8 (506)	<0.001
SES % (N) *				
Medium/high	92.2 (225)	85.2 (641)	86.9 (866)	
Low	7.8 (19)	14.8 (111)	13.1 (130)	0.005
DMF/T % (N) *				
0	80.7 (197)	65.0 (489)	68.9 (686)	
>0	19.3 (47)	35.0 (263)	31.1 (310)	<0.001
Orthodontics % (N) *				
No	76.2 (523)	78.4 (243)	76.9 (766)	
Yes	24.2 (59)	22.7 (171)	23.1 (230)	0.643
Coordination test ^~^ (Mean ± SD) ^++^				
Backward balancing	0.53 (±0.88)	0.34 (±1.05)	0.39 (±1.01)	0.006
One-leg-stand	0.69 (±0.70)	0.51 (±0.81)	0.55 (±0.79)	<0.001
Jumping side-to-side	0.24 (±1.18)	0.29 (±1.16)	0.28 (±1.16)	0.533

^++^*t*-test; * chi-square Test; SD, standard deviation; SES, socioeconomic status; ^~^ coordination tests: SDS transformed (−3 to 3).

**Table 2:** Association of dental caries experience (DMF/T) and SES, sex, age, orthodontic treatment, oral hygiene (plaque level) and coordination skills in children and adolescents of 10 to 18 years (Leipzig, Germany).

	DMF/T		Total	*p*-Value
	0	>0		
Total	68.9% (N = 686)	31.1% (N = 310)	100% (N = 996)	
Mean age (±SD) yrs. ^++^	12.47 (±1.99)	13.31 (±2.18)	12.73 (±2.09)	<0.001
Sex % (N) *				
Male	49.7 (341)	48.1 (149)	49.2 (490)	
Female	50.3 (345)	51.9 (161)	50.8 (506)	0.631
SES % (N) *				
Medium/high	90.4 (620)	79.4 (246)	86.9 (866)	
Low	9.6 (66)	20.6 (64)	13.1 (130)	<0.001
Orthodontics % (N) *				
No	76.2 (523)	78.4 (243)	76.9 (766)	
Yes	23.8 (163)	21.6 (67)	23.1 (230)	0.456
Oral Hygiene % (N) *				
Good	28.7 (197)	15.2 (47)	24.5 (244)	
Fair/Poor	71.3 (489)	84.8 (263)	75.5 (752)	<0.001
Coordination test ^~^ (Mean ± SD) ^++^				
Balancing backwards	0.45 (±0.99)	0.25 (±1.05)	0.39 (±1.01)	0.005
One-leg-stand	0.60 (±0.76)	0.45 (±0.84)	0.55 (±0.79)	0.009
Jumping side-to-side	0.31 (±1.15)	0.21 (1.20)	0.28 (±1.16)	0.180

^++^*t*-test; * chi-square Test; SD, standard deviation; SES, socioeconomic status; ^~^ coordination tests: SDS transformed (−3 to 3).

**Table 3:** Logistic regression analysis of dental caries experience (DMF/T)/Oral hygiene and SES, sex, age, orthodontic treatment and coordination skills in children and adolescents of 10 to 18 years (Leipzig, Germany).

		DMF/T			Oral Hygiene *			
	Odds Ratio	95%CI		*p*-Value	Odds Ratio	95%CI		*p*-Value
		Lowest	Highest			Lowest	Highest	
Age	1.25–1.26 ^§^	1.13–1.14 ^§^	1.38–1.40 ^§^	<0.001	0.90–0.91 ^§^	0.84–0.85 ^§^	0.97–0.98 ^§^	0.004–0.009 ^§^
Female gender	1.03	0.74	1.42–1.43 ^§^	0.855–0.878 ^§^	0.57	0.42	0.76–0.77 ^§^	0.001–0.008 ^§^
Low SES	1.65–1.86 ^§^	0.94–1.05 ^§^	2.92–3.23 ^§^	0.032–0.082 ^§^	2.18–2.58 ^§^	1.23–1.47 ^§^	3.98–4.70 ^§^	0.001–0.008 ^§^
Orthodontics ^#^	1.65–1.67 ^§^	0.93–0.95 ^§^	2.86–2.91 ^§^	0.072–0.081 ^§^	0.94–0.96 ^§^	0.67–0.68 ^§^	1.34–1.37 ^§^	0.785–0.829 ^§^
Plaque level ^+^								
2 ^+^	2.08–2.13 ^§^	1.37–1.39 ^§^	3.27–3.34 ^§^	<0.001	-	-	-	-
3 ^+^	6.88–7.14 ^§^	3.44–3.58 ^§^	14.08–14.56 ^§^	<0.001	-	-	-	-
One-leg-stand	0.81	0.66	0.99	0.037	0.78	0.63	0.96	0.018
Backward balancing	0.88	0.75	1.03	0.111	0.86	0.73	0.99	0.051
Jumping side-to-side	0.97	0.85	1.12	0.709	1.06	0.93	1.20	0.387

* Oral hygiene = good vs. fair/poor; ^§^ value depending on the type of coordination test in the analysis; ^#^ braces in the past for DMF/T and braces now for oral hygiene; ^+^ reference = good; 2 = fair; 3 = poor.

**Table 4:** Poisson regression analysis of dental caries experience (DMF/T) and SES, sex, age, orthodontic treatment, oral hygiene (plaque) and coordination skills in children and adolescents of 10 to 18 years (Leipzig, Germany).

		DMF/T		
	Exp (β)	95%CI		*p*-Value
		Lowest	Highest	
Age	1.22–1.23 ^§^	1.16–1.17 ^§^	1.29–1.30 ^§^	<0.001
Female gender	1.11–1.12 ^§^	0.93–0.94 ^§^	1.33–1.34 ^§^	0.216–0.245 ^§^
Low SES	2.31–2.50 ^§^	1.67–1.81 ^§^	3.21–3.46 ^§^	<0.001
Orthodontics past	1.45–1.49 ^§^	1.11–1.12 ^§^	1.92–1.93 ^§^	0.004–0.006 ^§^
Plaque ^+^				
2 ^+^	2.16–2.20 ^§^	1.64–1.67 ^§^	2.90–2.96 ^§^	<0.001
3 ^+^	4.35–4.57 ^§^	3.06–3.21 ^§^	6.24–6.54 ^§^	<0.001
One-leg-stand	0.82	0.74	0.91	<0.001
Backward balancing	0.94	0.87	1.02	0.132
Jumping side-to-side	0.94	0.88	1.02	0.125

^§^ Value depending on the type of coordination test in the analysis; SES, socioeconomic status; ^+^ Reference = good; 2 = fair; 3 = poor.

**Table 5:** Proportional odds model of oral hygiene (plaque level) and SES, sex, age, orthodontic treatment and coordination skills in children and adolescents of 10 to 18 years (Leipzig, Germany).

		Oral Hygiene *		
	Odds Ratio	95%CI		*p*-Value
		Lowest	Highest	
Age	0.92–0.93 ^§^	0.86–0.87 ^§^	0.98–0.99 ^§^	0.014–0.023 ^§^
Female gender	0.53–0.54 ^§^	0.41–0.41 ^§^	0.71–0.72 ^§^	<0.001
Low SES	2.11–2.48 ^§^	1.29–1.53 ^§^	3.44–4.03 ^§^	<0.001–0.002 ^§^
Orthodontics now	1.09–1.19 ^§^	0.79–0.80 ^§^	1.50–1.53 ^§^	0.528–0.614 ^§^
One-leg-stand	0.77	0.64	0.92	0.003
Backward balancing	0.86	0.75	0.99	0.037
Jumping side-to-side	1.00	0.89	1.13	0.935

* Oral hygiene = good vs. fair vs. poor; SES, socioeconomic status; ^§^ value depending on the type of coordination test in the analysis.

## References

[1] Müller L.K., Jungbauer G., Jungbauer R., Wolf M., Deschner J., Eick S. (2020). Biofilm and Orthodontic Therapy. Oral Biofilms.

[2] Thavarajah R., Kumar M., Mohandoss A.A., Vernon L.T. (2015). Drilling Deeper into tooth brushing skills: Is proactive interference an under-recognized factor in oral hygiene behavior change?. Curr. Oral Health Rep..

[3] Jackson S.L., Vann W.F., Kotch J.B., Pahel B.T., Lee J.Y. (2011). Impact of poor oral health on children’s school attendance and performance. Am. J. Public Health.

[4] Benjamin R.M. (2010). Oral health: The silent epidemic. Public Health Rep..

[5] Qadri G., Alkilzy M., Feng Y.-S., Splieth C. (2015). Overweight and dental caries: The association among German children. Int. J. Paediatr. Dent..

[6] Deutsche Arbeitsgemeinschaft für Jugendzahnpflege (DAJ) (2017). Epidemiologische Begleituntersuchungen zur Gruppenprophylaxe 2016. Bonn. https://www.daj.de/fileadmin/user_upload/PDF_Downloads/Epi_2016/Epi_final_BB1801_final.pdf.

[7] Thomson W.M., Poulton R., Milne B.J., Caspi A., Broughton J.R., Ayers K.M.S. (2004). Socioeconomic inequalities in oral health in childhood and adulthood in a birth cohort. Commun. Dent. Oral Epidemiol..

[8] Reisine S.T., Psoter W. (2001). Socioeconomic status and selected behavioral determinants as risk factors for dental caries. J. Dent. Educ..

[9] Schlueter N., Klimek J., Ganss C. (2013). Relationship between plaque score and video-monitored brushing performance after repeated instruction—A controlled, randomised clinical trial. Clin. Oral Investig..

[10] Jong-Lenters M.D., L’Hoir M., Polak E., Duijster D. (2019). Promoting parenting strategies to improve tooth brushing in children: Design of a non-randomised cluster-controlled trial. BMC Oral Health..

[11] Löe H. (2000). Oral hygiene in the prevention of caries and periodontal disease. Int. Dent. J..

[12] Inada E., Saitoh I., Yu Y., Tomiyama D., Murakami D., Takemoto Y., Morizono K., Iwasaki T., Yamasaki Y. (2015). Quantitative evaluation of toothbrush and arm-joint motion during tooth brushing. Clin. Oral Investig..

[13] Uenoyama A., Inada J. (1990). Muscle activities in the hand and arm during tooth brushing and the regulation of brushing movements by oral sensory perception. J. Osaka Dent. Univ..

[14] Kruis I. (2011). Advies Cariëspreventie: Praktische Handleiding Voor Wie Professionele Adviezen Geeft over Preventie Mondzorg. Zoetermeer. http://www.dentagon.nl/wp-content/uploads/2012/02/Advies-Cari%C3%ABspreventie-.pdf.

[15] Mahmoodi P., Salimi P., Ashtiyani R.D., Valaii N., Azarshab M., Shafizadeh N. (2014). Assessment of Fine Motor Skills and Tooth Brushing Skills in 5–6 Year Olds in Tehran. J. Dent. Res..

[16] Youcharoen K., Thomngam N., Aranya N., Wongphanthuset Y., Rungruangpattana M., Kaewsutha N. (2021). Plaque Removal Efficacy of Triple-Headed Toothbrush in 4–6-Year-Old Children: A Randomized Crossover Study. J. Int. Soc. Prev. Commun. Dent..

[17] Makuch A., Reschke K., Tiemann M., Mohokum M. (2021). Orales Gesundheitsverhalten—Ein wichtiges Feld der Prävention und Gesundheitsförderung. Prävention und Gesundheitsförderung: Mit 169 Abbildungen und 117 Tabellen.

[18] Robinson P.G., Deacon S.A., Deery C., Heanue M., Walmsley A.D., Worthington H.V., Glenny A.-M. (2005). Manual versus powered toothbrushing for oral health. Cochrane Database Syst. Rev..

[19] Poyato-Ferrera M., Segura-Egea J.J., Bullón-Fernández P. (2003). Comparison of modified Bass technique with normal toothbrushing practices for efficacy in supragingival plaque removal. Int. J. Dent. Hyg..

[20] Werner H., Hakeberg M., Dahlström L., Eriksson M., Sjögren P., Strandell A., Svanberg T., Svensson L., Wide Boman U. (2016). Psychological Interventions for Poor Oral Health: A Systematic Review. J. Dent. Res..

[21] Hitz Lindenmüller I., Lambrecht J., Surber C. (2011). Oral Care. Topical Applications and the Mucosa: 27 Tables.

[22] Piek J.P., Baynam G.B., Barrett N.C. (2006). The relationship between fine and gross motor ability, self-perceptions and self-worth in children and adolescents. Hum. Mov. Sci..

[23] Cantell M.H., Smyth M.M., Ahonen T.P. (1994). Clumsiness in Adolescence: Educational, Motor, and Social Outcomes of Motor Delay Detected at 5 Years. Adapt. Phys. Act. Q..

[24] Schmidt J., Guder U., Kreuz M., Löffler M., Kiess W., Hirsch C., Ziebolz D., Haak R. (2018). aMMP-8 in correlation to caries and periodontal condition in adolescents-results of the epidemiologic LIFE child study. Clin. Oral. Investig..

[25] Poulain T., Vogel M., Sobek C., Hilbert A., Körner A., Kiess W. (2019). Associations between Socio-Economic Status and Child Health: Findings of a Large German Cohort Study. Int. J. Environ. Res. Public Health.

[26] Quante M., Hesse M., Döhnert M., Fuchs M., Hirsch C., Sergeyev E., Casprzig N., Geserick M., Naumann S., Koch C. (2012). The LIFE child study: A life course approach to disease and health. BMC Public Health.

[27] Elger W., Kiess W., Körner A., Schrock A., Vogel M., Hirsch C. (2019). Influence of overweight/obesity, socioeconomic status, and oral hygiene on caries in primary dentition. J. Investig. Clin. Dent..

[28] Bös K., Worth A., Opper E., Oberger J., Rohman N., Wagner M. (2009). Motorik-Modul: Eine Studie zur motorischen Leistungsfähigkeit und körperlich-sportlichen Aktivität von Kindern und Jugendlichen in Deutschland. Abschlussbericht zum Forschungsprojekt.

[29] Wagner M.O., Bös K., Jekauc D., Karger C., Mewes N., Oberger J., Reimers A.K., Schlenker L., Worth A., Woll A. (2014). Cohort profile: The Motorik-Modul Longitudinal Study: Physical fitness and physical activity as determinants of health development in German children and adolescents. Int. J. Epidemiol..

[30] Opper E., Worth A., Wagner M., Bös K. (2007). Motorik-Modul (MoMo) im Rahmen des Kinder- und Jugendgesundheitssurveys (KiGGS). Motorische Leistungsfähigkeit und körperlich-sportliche Aktivität von Kindern und Jugendlichen in Deutschland. [The module “Motorik” in the German Health Interview and Examination Survey for Children and Adolescents (KiGGS). Motor fitness and physical activity of children and young people]. Bundesgesundheitsblatt Gesundh. Gesundh..

[31] Woll A., Kurth B.-M., Opper E., Worth A., Bös K. (2011). The ‘Motorik-Modul’ (MoMo): Physical fitness and physical activity in German children and adolescents. Eur. J. Pediatr..

[32] Schilling W., Baedke D. (1980). Screening-Test fuer den motorischen Bereich bei der Einschulung. Motorik.

[33] Kiphard E.J., Schilling F. (2007). Körperkoordinationstest für Kinder: 2. Überarb.

[34] Möller S., Poulain T., Körner A., Meigen C., Jurkutat A., Vogel M., Wessela S., Hiemisch A., Grafe N., Kiess W. (2021). Motor skills in relation to body-mass index, physical activity, TV-watching, and socioeconomic status in German four-to-17-year-old children. PLoS ONE.

[35] Winkler J., Stolzenberg H. (2009). Adjustierung des Sozialen-Schicht-Index für die Anwendung im Kinder- und Jugendgesundheitssurvey (KiGGS).

[36] Greene J.C., Vermillion J.R. (1964). The Simplified Oral Hygiene Index. J. Am. Dent. Assoc..

[37] R Core Team (2022). R: A Language and Environment for Statistical Computing.

[38] Brant R. (1990). Assessing Proportionality in the Proportional Odds Model for Ordinal Logistic Regression. Biometrics.

[39] Tangnuntachai N., Smutkeeree A., Jirarattanasopha V., Leelataweewud P. (2021). Visual pedagogy-guided toothbrushing training to enhance fine motor skills in individuals with intellectual disabilities and impaired fine motor skills. Special care in dentistry: Official publication of the American Association of Hospital Dentists, the Academy of Dentistry for the Handicapped, and the American Society for Geriatric Dentistry. Spec. Care Dent..

[40] Diamanti I., Berdouses E.D., Kavvadia K., Arapostathis K.N., Polychronopoulou A., Oulis C.J. (2021). Oral hygiene and periodontal condition of 12- and 15-year-old Greek adolescents. Socio-behavioural risk indicators, self- rated oral health and changes in 10 years. Eur. J. Paediatr. Dent..

[41] Kudirkaite I., Lopatiene K., Zubiene J., Saldunaite K. (2016). Age and gender influence on oral hygiene among adolescents with fixed orthodontic appliances. Stomatologija.

[42] Jordan A.R., Micheelis W., Cholmakow-Bodechtel C. (2016). Fünfte Deutsche Mundgesundheitsstudie (DMS V).

[43] Vadiakas G., Oulis C.J., Tsinidou K., Mamai-Homata E., Polychronopoulou A. (2012). Oral hygiene and periodontal status of 12 and 15-year-old Greek adolescents. A national pathfinder survey. Eur. Arch. Paediatr. Dent..

[44] Das U.M., Singhal P. (2009). Tooth brushing skills for the children aged 3–11 years. J. Indian Soc. Pedod. Prev. Dent..

[45] Meyer-Lückel H., Paris S., Ekstrand K.R., Alkilzy M., Effenberger S., Lackner C. (2012). Karies: Wissenschaft und Klinische Praxis.

[46] Reich E., Lussi A., Newbrun E. (1999). Caries-risk assessment. Int. Dent. J..

[47] Schwendicke F., Dörfer C.E., Schlattmann P., Foster Page L., Thomson W.M., Paris S. (2015). Socioeconomic inequality and caries: A systematic review and meta-analysis. J. Dent. Res..

[48] Lancet T. (2009). Oral health: Prevention is key. Lancet.

[49] Choi Y.Y. (2020). Relationship between orthodontic treatment and dental caries: Results from a national survey. Int. Dent. J..

[50] Cave V., Hutchison C. (2020). Does orthodontic treatment affect caries levels?. Evid. Based Dent..

[51] Walsh L.J., Healey D.L. (2019). Prevention and caries risk management in teenage and orthodontic patients. Aust. Dent. J..

